# Pre-existing Pulmonary Diseases and Survival in Patients With Stage-dependent Lung Adenocarcinoma: A STROBE-compliant Article

**DOI:** 10.1097/MD.0000000000002987

**Published:** 2016-03-11

**Authors:** Zhi-Hong Jian, Jing-Yang Huang, Oswald Ndi Nfor, Kai-Ming Jhang, Wen-Yuan Ku, Chien-Chang Ho, Chia-Chi Lung, Hui-Hsien Pan, Yu-Chiu Liang, Ming-Fang Wu, Yung-Po Liaw

**Affiliations:** From the Department of Public Health and Institute of Public Health (Z-HJ, J-YH, ONN, K-MJ, W-YK, C-CL, Y-PL); School of Medicine, Chung Shan Medical University (H-HP, M-FW); Department of Family and Community Medicine (C-CL, Y-PL); Department of Pediatrics (H-HP); Divisions of Medical Oncology and Pulmonary Medicine, Chung Shan Medical University Hospital, Taichung City (M-FW); Department of Neurology, Changhua Christian Hospital, Changhua (K-MJ); Department of Physical Education, Fu Jen Catholic University, New Taipei City (C-CH); and College of Humanities and Social Sciences and Taipei Medical University (Y-CL), Taipei City, Taiwan.

## Abstract

Asthma, chronic obstructive pulmonary disease (COPD), and pulmonary tuberculosis (TB) are common lung diseases associated with lung cancer mortality. This study evaluated sex disparities in pre-existing pulmonary diseases and stage-dependent lung adenocarcinoma survival.

Patients newly diagnosed with lung adenocarcinoma between 2003 and 2008 were identified using the National Health Insurance Research Database and Cancer Registry. Cases with lung adenocarcinoma were followed until the end of 2010. Survival curves were estimated by the Kaplan–Meier method. Cox proportional-hazard regression was used to calculate the hazard ratio (HR) of pre-existing asthma, COPD, and/or TB, and to estimate all-cause mortality risk in patients with different stages of lung adenocarcinoma.

A total of 14,518 cases were identified with lung adenocarcinoma. Specifically, among men, the HRs for TB were 1.69 (95% confidence interval [CI], 1.10–2.58), 1.48 (95% CI, 1.14–1.93), and 1.27 (95% CI, 1.08–1.49) for individuals with stage I + II, III, and IV diseases, respectively. The HRs for asthma were 1.41 (95% CI, 1.00–1.99) in women with stage I + II and 1.14 (95% CI, 1.04–1.26) in men with stage IV disease. For pulmonary disease combinations in men, the HRs were 1.45 (95% CI, 1.12–1.89) for asthma + COPD + TB, 1.35 (95% CI, 1.12–1.63) for COPD + TB, 1.28 (95% CI, 1.01–1.63) for TB, and 1.15 (95%CI, 1.04–1.27) for asthma + COPD, respectively. For women with stage I + II disease, the HR was 6.94 (95% CI, 2.72–17.71) for asthma + COPD + TB.

Coexistence of pre-existing pulmonary diseases increased mortality risk in men with adenocarcinoma. TB is at elevated risk of mortality among men with different stages of adenocarcinoma. Asthmatic women with early-stage adenocarcinoma had increased risk of mortality.

## Abstract 2

Supplemental Digital Content is available in the text

## INTRODUCTION

Lung cancer is the leading cause of cancer death. The 5-year survival rates by stage have been reported as 60.7% for stage I, 36.3% for stage II, 13.3% for stage III, and 4.9% for stage IV, repsectively.^1^ Adenocarcinoma is the most common histologic type and accounts for approximately 47.3 % of all lung cancers in Taiwan.^2^ In addition to stages, the prognosis of lung cancer largely depends on the performance status of patient, sex, and comorbidities.^3–6^ Lung cancer is associated with age and smoking, which are strongly associated with comorbidities.^7–9^ More than half of patients with lung cancer have been reported with 3 or more comorbidities.^10^ Several studies have found that approximately 29% to 57% of predominantly stage I nonsmall cell lung cancer (NSCLC) patients died of competing causes without evidence of lung cancer recurrence or progression.^11,12^

Asthma, chronic obstructive pulmonary disease (COPD), and pulmonary tuberculosis (TB) are the most common lung comorbidities. Inflammation and lung function decline are the main pathophysiological features of COPD. Zhang et al^13^ reported that COPD was induced in a rat after it was exposed to cigarette smoke for 8 weeks and injected with lipopolysaccharide twice. They also reported increases in tumor necrosis factor-α and interleukin-1β levels in bronchoalveolar lavage fluid, orexin A, and its receptor level, and also mRNA expressions of orexin A receptor in lung tissues. Asthma,^14^ COPD,^15^ and TB^16^ lead to airway inflammation and can also be associated with the increased risk of lung cancer. Among patients with lung adenocarcinoma, the prevalence rates of asthma, COPD, and TB have been reported as 10.7%, 21.4%, and 3.2%, respectively.^17^ Tammemagi et al^10^ found that asthma, COPD, and TB were independent predictors of lung cancer survival in a cohort of 1155 patients. Jian et al^17^ reported that death due to lung adenocarcinoma was significantly high in men with coexisting pulmonary diseases. However, they did not investigate the impact of coexisting asthma, COPD, and TB on stage-dependent survival. Furthermore, smoking is 10 times more prevalent in Taiwanese men than women.^18^ This may have influenced the sex-based differences in survival. The aim of this study was to investigate the relationship between pre-existing pulmonary diseases (asthma, COPD, and/or TB) and survival in patients with adenocarcinoma by sex and stage.

## METHODS

### Ethics Statement

The Institutional Review Board of the Chung-Shan Medical University Hospital, Taiwan, approved this study. Individual informed consent was waived because the source data were deidentified.

### Data Source

The analytical data used in this study were retrieved from multiple datasets which included the National Health Insurance Research Database (NHIRD), Taiwan Cancer Registry Database (TCRD), and National Death Registry Database (NDRD). Detailed information about the datasets has been described previously.^17,19,20^ The coverage rate is about 99%.

The NHIRD contain demographic information, records of clinical visits and hospitalizations, and also disease coding, medical care, cost, and institutions. Inclusion criteria were a diagnosis of lung cancer (International Classification of Diseases, Ninth Revision, Clinical Modification [ICD-9-CM] code 162) in individuals 20 years of age and older during the period 2003 to 2008. The index date was defined as the date of lung cancer diagnosis. The exclusion criteria were lung cancer before 2002, unknown sex, and incomplete registry data.

The TCRD was used to confirm the histologic types of lung cancer. It contained information about diagnosis date, cancer site, clinical stage, and histology. Lung cancers were coded by ICD-9-CM 162 or ICD 10 C34.0, C34.1, C34.2, C34.3, C34.8, and C34.9 in TCRD. Morphological diagnoses were made using the ninth revision of the International Classification of Diseases for Oncology, based primarily on codes 80503, 81402, 81403, 81413, 81433, 82113, 82503, 82513, 82523, 82553, 82603, 83103, 83233, 84603, 84803, 84813, 84903, and 85003 for adenocarcinoma.

The NDRD contained information on survival, date of death, and cause of death. The lung cancer patients were linked to the NDRD to obtain follow-up information (such as person-months of follow-up, death, and survival time) available until the study end in 2010.

### Exposed Variables

The definition of comorbidity was defined when those ICD-9 CM coding existed 2 years before the index date. Diagnoses of pulmonary diseases and other comorbidities were confirmed by either 2 outpatient consultations or 1 admission in a year. Pulmonary diseases and other comorbidities were as follows: asthma (ICD-9-CM: 493), COPD (ICD-9-CM: 490, 491, 492, 494, and 496), TB (ICD-9-CM: 010–012, and 137.0), chronic renal disease (ICD-9-CM: 585 and 586), diabetes mellitus (ICD-9-CM: 250), hyperlipidemia (ICD-9-CM: 272), and smoking-related cancers (ICD-9-CM: 140–150, 157, 160–161, and 189).

Medications such as statins,^21^ corticosteroids,^22^ and aspirin^23^ have been associated with lung cancer. We identified patients who were treated with inhaled and oral corticosteroids, statins, and aspirin before the index date.

### Statistical Analysis

All analyses were conducted using the SAS 9.3 software (SAS Institute, Cary, NC). The effect of predictor variables on survival was evaluated at the univariate level using Kaplan–Meier survival plots and log-rank test, and at multivariate levels using hazard ratios (HRs) and associated 95% confidence intervals (CIs) by Cox proportional-hazards regression modeling. Three models were used to analyze the effect of combinations of pulmonary diseases on the overall mortality risk of lung adenocarcinoma stratified by sex and stage, and adjusted for the baseline covariates. Model 1 consists of 3 pulmonary diseases, model 2 contains pulmonary disease combinations, and Model 3 is a count of pulmonary diseases. All comparisons with a *P* value <0.05 were considered to be statistically significant.

## RESULTS

**FIGURE 1:**
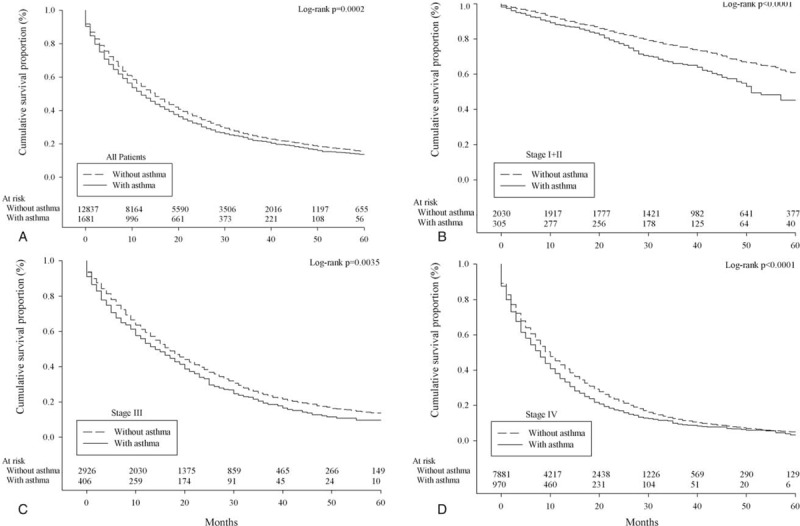
Survival of lung adenocarcinoma patients with/without asthma according to overall stage grouping (A, all patients; B, stage I+II; C, stage III; D, stage IV).

**FIGURE 2:**
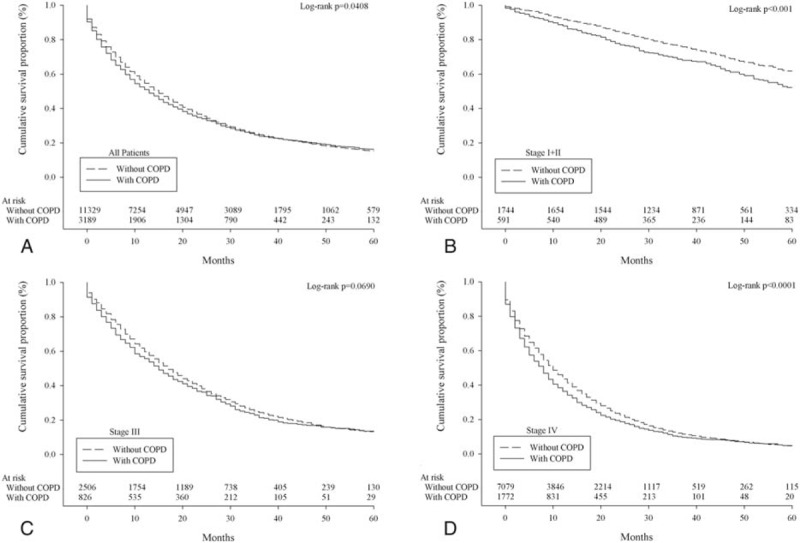
Survival of lung adenocarcinoma patients with/without chronic obstructive pulmonary disease according to overall stage grouping (A, all patients; B, stage I + II; C, stage III; D, stage IV).

**FIGURE 3:**
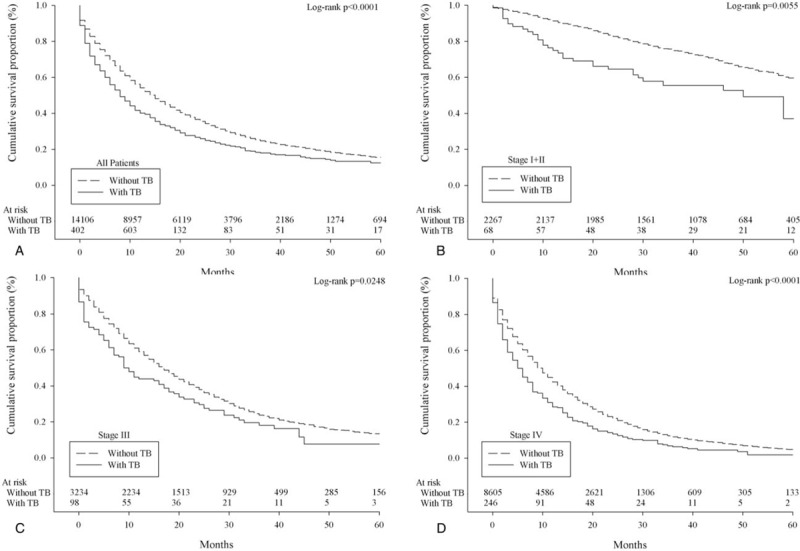
Survival of lung adenocarcinoma patients with/without pulmonary tuberculosis according to overall stage grouping (A, all patients; B, stage I + II; C, stage III; D, stage IV).

A total of 14,518 patients were diagnosed with lung adenocarcinoma between 2003 and 2008. Detailed characteristics of the study population, and the distribution and associations of comorbidities are presented in the Supplementary Table S1 and Figure S1. Kaplan–Meier survival plots for asthma, COPD, and TB by stage, which are univariate unadjusted depictions, are presented in Figure 1, Figure 2, and Figure 3, respectively. Patients with asthma, COPD, and TB were at greater risk of mortality from lung adenocarcinoma.

The HRs of mortality were 1.09 (95% CI, 1.02–1.16) for asthma, 0.98 (95%CI, 0.93–1.02) for COPD, and 1.24 (95%CI, 1.11–1.39) for TB after adjusting for the baseline covariates (see Table S2 Supplemental Content, which illustrates the HRs of all-cause mortality in patients with lung adenocarcinoma).

**FIGURE 4:**
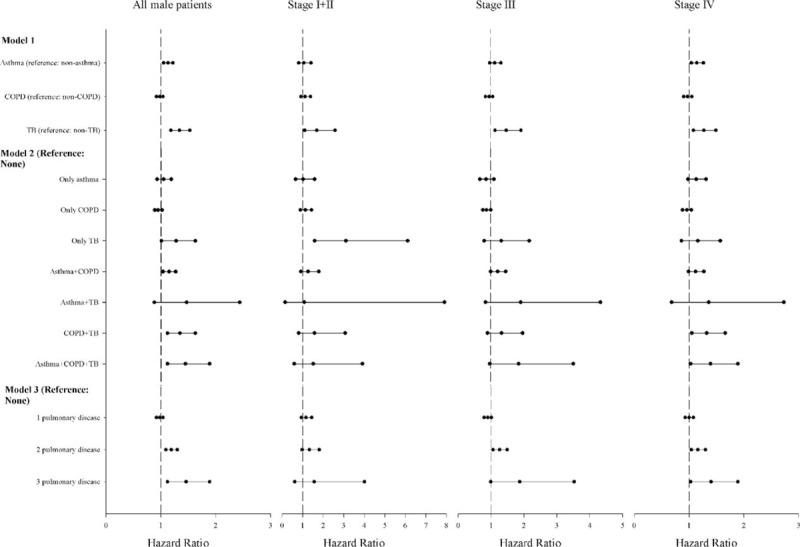
Multivariable analyses in men. The hazard ratios of mortality for pulmonary diseases in patients with lung adenocarcinoma by stage after adjusting for age, low income, surgery, comorbidities, geographical area, urbanization, and medications. Model 1—a model containing three pulmonary diseases; model 2—a model containing pulmonary disease combinations; and model 3—a count of pulmonary diseases. COPD = chronic obstructive pulmonary disease, TB = tuberculosis.

Figure 4 shows the pre-existing pulmonary diseases and the risk of death from lung adenocarcinoma according to models and stage in men. In model 1, TB was responsible for the increased mortality with HRs of 1.69 (95% CI, 1.10–2.58), 1.48 (95% CI, 1.14–1.93), and 1.27 (95% CI, 1.08–1.49) for patients with stage I + II, III, and IV disease, respectively (see Supplementary material, Table S3). The HR for asthma was 1.14 (95% CI, 1.04–1.26) in men with stage IV disease. In model 2, for all male patients, the HRs were higher among individuals with asthma + COPD + TB (HR 1.45; 95% CI, 1.12–1.89), COPD + TB (HR 1.35; 95% CI, 1.12–1.63), TB (HR 1.28; 95% CI, 1.01–1.63), and asthma + COPD (HR 1.15; 95% CI, 1.04–1.27). For stage IV cancer, the HRs were 1.39 (95% CI, 1.03–1.89) and 1.32 (95% CI, 1.05–1.66) for patients with asthma + COPD + TB and COPD + TB, respectively. In model 3, an increase in the number of pulmonary diseases was associated with increased risk of mortality, particularly in men. For example, the HRs were 1.19 (95% CI, 1.09–1.30) and 1.46 (95% CI, 1.12–1.89) in men with 2 and 3 pulmonary diseases, respectively. For stage IV cancer, the HRs were 1.16 (95% CI, 1.04–1.30) and 1.40 (95% CI, 1.03–1.89) in men with 2 and 3 pulmonary diseases, respectively.

**FIGURE 5:**
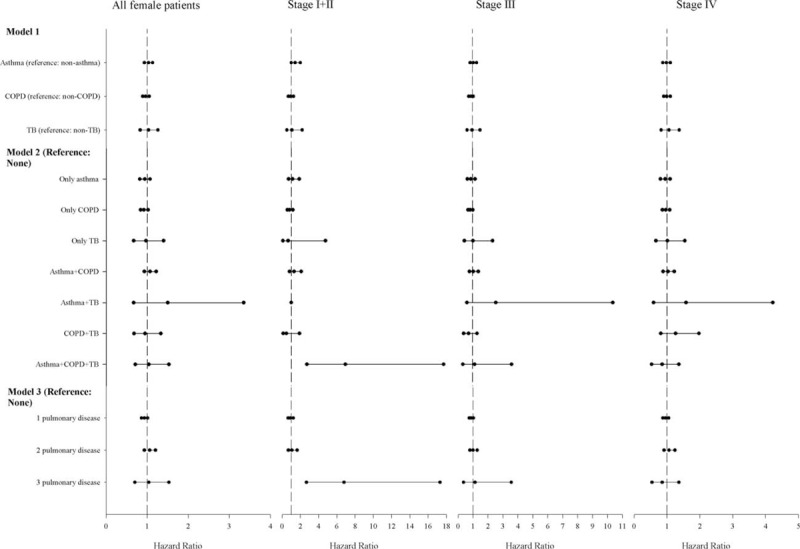
Multivariable analyses in women. The hazard ratios of mortality for pulmonary diseases in patients with lung adenocarcinoma by stage after adjusting for age, low income, surgery, comorbidities, geographical area, urbanization, and medications. Model 1—a model containing three pulmonary diseases; model 2—a model containing pulmonary disease combinations; and model 3—a count of pulmonary diseases. COPD = chronic obstructive pulmonary disease, TB = tuberculosis.

Figure 5 illustrates the HRs of pre-existing pulmonary diseases in women by model and stage. In model 1, the HR for asthma was 1.41 (95% CI, 1.00–1.99) in patients with stage I + II (also see Supplementary material, Table S4). In model 3, and for stage I + II, the HR was 6.79 (95% CI, 2.66–17.33) for individuals with 3 pulmonary diseases.

## DISCUSSION

Most patients with lung adenocarcinoma consult with their physicians in the late stage of disease.^17^ Many patients with lung cancer have concurrent comorbidities that significantly affect their overall health. Tammemagi et al^10^ analyzed the risk factors of lung cancer survival and found that stage explained 25.4% of the survival variation, whereas comorbidities explained 6.1%, treatments 9.2%, and age 3.7%. In general, we found that TB and pulmonary diseases were associated with increased risk of mortality in men with adenocarcinoma. Asthma was found to significantly increase mortality risk in women with early stage, and men with stage IV adenocarcinoma.

Vesterinen et al^24^ analyzed the survival rates of lung cancer in patients with a preceding diagnosis of bronchial asthma and matched nonasthma controls during 1970 to 1989. They found no significant differences in the prognosis of lung cancer (corresponding 5-year survival rates were 8.5% and 8.1%). With treatment advances that increase the life expectancy of lung cancer patients, deaths may result from competing causes, whereas comorbidities may lower prognosis.^25^ Brown et al^26^ analyzed data from 9087 adults aged 30 to 75 years from the Second National Health and Nutrition Examination Survey (NHANES II) and NHANES II Mortality Study. Among 6144 nonsmokers, the adjusted relative risk of asthma for lung cancer mortality was 3.54 (95% CI, 1.93–6.42). In a retrospective cohort study, the risk of mortality was significantly increased only in men with asthma (HR 1.20; 95% CI, 1.10–1.30).^17^ Stage and comorbidities are both critical for lung cancer survival. In this study, asthma was specifically associated with the mortality in men with stage IV and women with stage I + II diseases.

The impact of COPD on mortality of lung cancer remains controversial, hence additional information is required. In a retrospective chart review involving 442 patients with stage IA lung cancer, COPD was associated with increased risk of overall mortality (HR 1.96; 95% CI, 1.14–3.36) and tumor recurrence (HR 2.07; 95% CI, 1.18–3.64).^27^ Izquierdo et al^28^ analyzed 324 cases with advanced lung cancer (stages IIIB and IV) who were receiving standard care and concluded that COPD does not have a significant deleterious impact on overall survival (HR 1.20; 95% CI, 0.83–1.50). However, the impact of COPD on the mortality from adenocarcinoma has not yet been investigated. Our findings showed that COPD specifically did not increase mortality risk in patients with different stages of adenocarcinoma.

In Hong Kong, TB has been associated with death due to lung cancer in older persons. The adjusted HRs were 2.81 (95% CI, 1.45–5.42) for nonsmokers and 1.76 (95% CI, 1.13–2.72) for smokers.^29^ However, this study did not investigate lung cancer by stage, histologic type, and surgical treatment. Chang et al^30^ analyzed 6073 epidermal growth factor receptor-tyrosine kinase inhibitor (EGFR-TKI) responder and 2192 EGFR-TKI nonresponder. Their study results showed that male patients with history of pulmonary TB had a poor EGFR-TKI response and 1-year progression-free survival of lung cancer. This is consistent with our findings.

Asthma and COPD may coexist in the same patients. They have been associated with increased systemic inflammation, mortality, and healthcare utilization than those with asthma or COPD alone.^31–33^ Inghammar et al^34^ analyzed 115,867 patients from the Swedish Inpatient Register and found that COPD patients with active TB had a 2-fold (HR 2.2; 95% CI, 1.2–4.1) increased risk of death compared with the controls. Moreover, there are several reports about the association between chronic inflammation and EGFR mutation. Oxidant-induced goblet cell metaplasia in human airway epithelium (such as asthma and chronic bronchitis) leads to EGFR activation.^35^ TB increases the expression of epiregulin which is a marker of advanced disease in NSCLC patients and confers invasive properties on EGFR-mutant cells.^36,37^ A recent retrospective study conducted in Taiwan showed that patients with lung adenocarcinoma who had either scar cancers or old TB lesions had a higher probability of having EGFR mutations, especially exon 19 deletions.^38^ Nevertheless, these studies emphasized that inflammation may cause EGFR mutations.

Our study results showed that coexisting pulmonary diseases affected more men than women, and were responsible for mortality in patients with lung adenocarcinoma. Because a ten-fold difference has been shown in the smoking prevalence between men and women in Taiwan,^18^ this might have driven the differences observed in lung adenocarcinoma mortality. Continued smoking after lung cancer diagnosis may worsen treatment efficacy, quality of life, overall survival, and can also increase the risk of secondary primary tumors.^39^ It can also increase the risk of tumor recurrence (HR 1.86; 95% CI, 1.01–3.41) in early-stage NSCLC.^40^ Hazard from exposure to fumes from cocking oil explained the majority of the attributable fraction (47.7%) of female adenocarcinoma.^41^ In addition, sex hormones are central to these differences, which may either contribute to the pathogenesis of disease or serve as protective factors.^42^ Female sex exerted a positive effect on disease-related survival of NSCLC patients receiving surgical resection irrespective of the histological subtype and stage.^43^ Estrogen receptor-β is more frequently expressed in lung tissues of women with NSCLC and is associated with degree of differentiation, lymph node metastasis, and survival.^44^ Overexpression of estrogen receptor -β expression correlates with EGFR mutations, good tumor differentiation, and an increasing disease-free survival in patients with EGFR mutations in adenocarcinoma.^45^ It has been reported that there are survival advantages in female patients in response to EGFR inhibitors and antiangiogenesis agents.^46,47^

This study has several strengths. First, compared with our previous study,^17^ we included “stage-specific” criteria to evaluate the effect of pulmonary diseases on survival in patients with adenocarcinoma. Second, it is a retrospective cohort study with a large sample size and long follow-up. This minimizes any concerns over reverse causality. Third, we included cases of asthma, COPD, and TB diagnosed 2 years before diagnosis of lung adenocarcinoma, hence the probability of misclassifying was minimized. Nevertheless, this study has several limitations. First, possible prognostic factors, such as performance status, visceral pleural invasion, and lymphovascular invasion, were not available. Second, smoking is a common risk for lung cancer and COPD, and pack-years of smoking is critical. However, information on smoking is not available in the multiple databases used in this study.

In conclusion, TB can have a deleterious prognostic impact on survival in men with different stages of adenocarcinoma. Coexisting pulmonary diseases also conferred a higher risk of mortality. Pre-existing asthma was associated with increased risk of mortality in women with early-stage adenocarcinoma.

## Supplementary Material

Supplemental Digital Content
