# Supplementary material for: Pre-existing Pulmonary Diseases and Survival in Patients With Stage-dependent Lung Adenocarcinoma: A STROBE-compliant Article
Source: Medicine (Baltimore). 2016 Mar 11;95(10):e2987. doi: 10.1097/MD.0000000000002987 (PMC4998887; doi:10.1097/MD.0000000000002987)
Supplement: Supplemental Digital Content [file medi-95-e02987-s001.docx]

| **TABLE S1.** Characteristics of Patients with Lung Adenocarcinoma (2003-2008) and All-Cause Mortality Rate (2003-2010). | | | | |
| --- | --- | --- | --- | --- |
|  | Duration (person-months) | No. of Deaths | Mortality rate  (per 100 person-months)  (95% C.I.) | Rate ratio  (95% C.I.) |
| Pulmonary diseases |  |  |  |  |
| Asthma |  |  |  |  |
| No (N= 12837) | 270734 | 10147 | 3.748 (3.747-3.749) | 1 |
| Yes (N=1681) | 31334 | 1348 | 4.302 (4.296-4.308) | 1.148 (1.146-1.149) |
| COPD |  |  |  |  |
| No (N= 11329) | 239562 | 9001 | 3.757 (3.756-3.758) | 1 |
| Yes (N= 3189) | 62506 | 2494 | 3.99 (3.987-3.993) | 1.062 (1.061-1.063) |
| TB |  |  |  |  |
| No (N= 14106) | 295127 | 11148 | 3.778 (3.777-3.778) | 1 |
| Yes (N= 412) | 6941 | 347 | 4.999 (4.971-5.027) | 1.324 (1.316-1.331) |
| Sex |  |  |  |  |
| Male (N= 8259) | 152589 | 6877 | 4.507 (4.505-4.508) | 1 |
| Female (N= 6259) | 149479 | 4618 | 3.089 (3.088-3.091) | 0.686 (0.685-0.686) |
| Age diagnosed with lung adenocarcinoma |  |  |  |  |
| <40 (N= 445) | 10512 | 342 | 3.253 (3.235-3.272) | 1 |
| 40-59 (N= 5101) | 125835 | 3696 | 2.937 (2.936-2.939) | 0.903 (0.898-0.908) |
| ≧60 (N= 8972) | 165721 | 7457 | 4.500 (4.498-4.501) | 1.383 (1.375-1.391) |
| Low income |  |  |  |  |
| No (N= 14234) | 297166 | 11242 | 3.783 (3.783-3.784) | 1 |
| Yes (N= 284) | 4902 | 253 | 5.161 (5.121-5.201) | 1.364 (1.354-1.375) |
| Stage |  |  |  |  |
| I (N= 1995) | 83138 | 607 | 0.73 (0.728-0.732) | 1 |
| II (N= 340) | 11726 | 166 | 1.416 (1.399-1.432) | 1.939 (1.915-1.963) |
| III (N= 3332) | 73136 | 2702 | 3.695 (3.692-3.697) | 5.060 (5.043-5.077) |
| IV (N= 8851) | 134068 | 8020 | 5.982 (5.981-5.984) | 8.193 (8.167-8.22) |
| Surgery |  |  |  |  |
| No (N= 11562) | 187520 | 10373 | 5.532 (5.531-5.533) | 1 |
| Yes (N= 2956) | 114548 | 1122 | 0.979 (0.978-0.981) | 0.177 (0.177-0.177) |
| Comorbidities |  |  |  |  |
| Diabetes |  |  |  |  |
| No (N= 12036) | 255392 | 9489 | 3.715 (3.715-3.716) | 1 |
| Yes (N= 2482) | 46676 | 2006 | 4.298 (4.293-4.302) | 1.157 (1.156-1.158) |
| Hyperlipidemia |  |  |  |  |
| No (N= 11393) | 234458 | 9185 | 3.918 (3.917-3.918) | 1 |
| Yes (N=3125) | 67610 | 2310 | 3.417 (3.414-3.42) | 0.872 (0.871-0.873) |
| Chronic renal disease |  |  |  |  |
| No (N= 14144) | 295583 | 11181 | 3.783 (3.782-3.783) | 1 |
| Yes (N= 374) | 6485 | 314 | 4.842 (4.812-4.872) | 1.280 (1.272-1.288) |
| Smoking related cancers |  |  |  |  |
| No (N= 14089) | 292197 | 11160 | 3.819 (3.819-3.820) | 1 |
| Yes (N= 429) | 9871 | 335 | 3.394 (3.374-3.414) | 0.889 (0.883-0.894) |
| Medication use prior to adenocarcinoma diagnosis |  |  |  |  |
| Inhaled corticosteroids |  |  |  |  |
| No (N= 13892) | 290963 | 10974 | 3.771 (3.771-3.772) | 1 |
| Yes (N= 626) | 11105 | 521 | 4.692 (4.674-4.709) | 1.244 (1.239-1.249) |
| Oral corticosteroids |  |  |  |  |
| No (N= 11147) | 239794 | 8866 | 3.697 (3.696-3.698) | 1 |
| Yes (N= 3371) | 62274 | 2629 | 4.222 (4.219-4.225) | 1.142 (1.141-1.143) |
| Aspirin |  |  |  |  |
| No (N= 11597) | 249930 | 9089 | 3.637 (3.636-3.637) | 1 |
| Yes (N= 2921) | 52138 | 2406 | 4.615 (4.611-4.619) | 1.269 (1.268-1.270) |
| Statin |  |  |  |  |
| No (N=13168) | 273298 | 10476 | 3.833 (3.832-3.834) | 1 |
| Yes (N= 1350) | 28770 | 1019 | 3.542 (3.535-3.549) | 0.924 (0.922-0.926) |
| Geographical area |  |  |  |  |
| Taipei (N= 4757) | 107319 | 3599 | 3.354 (3.352-3.356) | 1 |
| North (N= 1639) | 33582 | 1306 | 3.889 (3.883-3.895) | 1.160 (1.158-1.161) |
| Central (N= 2786) | 57105 | 2222 | 3.891 (3.888-3.894) | 1.160 (1.159-1.161) |
| South (N= 2706) | 52204 | 2210 | 4.233 (4.23-4.237) | 1.262 (1.261-1.264) |
| Kaohsiung-Pingtung (N= 2240) | 45136 | 1828 | 4.050 (4.045-4.054) | 1.208 (1.206-1.209) |
| East (N= 390) | 6722 | 330 | 4.909 (4.88-4.939) | 1.464 (1.455-1.473) |
| Urbanization |  |  |  |  |
| Urban(N= 7757) | 173619 | 5913 | 3.406 (3.405-3.407) | 1 |
| Sub-urban(N= 4691) | 90320 | 3834 | 4.245 (4.243-4.247) | 1.246 (1.246-1.247) |
| Rural(N= 2070) | 38129 | 1748 | 4.584 (4.579-4.59) | 1.346 (1.344-1.348) |
| CI, confidence interval; COPD, chronic obstructive pulmonary disease; TB, tuberculosis. | | | | |

| **TABLE S2.** Cox Proportional Model to Estimate the Hazard Ratios for All-Cause Mortality in Patients with Lung Adenocarcinoma | | | |
| --- | --- | --- | --- |
|  | HR | 95% C.I. | p-value |
| Pulmonary diseases |  |  |  |
| Asthma |  |  |  |
| No | 1 |  |  |
| Yes | **1.09** | 1.02-1.16 | 0.006 |
| COPD |  |  |  |
| No | 1 |  |  |
| Yes | 0.98 | 0.93-1.02 | 0.317 |
| TB |  |  |  |
| No | 1 |  |  |
| Yes | **1.24** | 1.11-1.39 | <0.001 |
| Sex |  |  |  |
| Female | 1 |  |  |
| Male | 1.33 | 1.28-1.38 | <0.001 |
| Age diagnosed with lung adenocarcinoma |  |  |  |
| <40 | 1 |  |  |
| 40-59 | 0.96 | 0.86-1.07 | 0.469 |
| ≧60 | 1.28 | 1.15-1.43 | <0.001 |
| Low income |  |  |  |
| No | 1 |  |  |
| Yes | 1.18 | 1.04-1.33 | 0.011 |
| Stage |  |  |  |
| I | 1 |  |  |
| II | 1.74 | 1.46-2.07 | <0.001 |
| III | 2.73 | 2.48-3.01 | <0.001 |
| IV | 3.88 | 3.53-4.27 | <0.001 |
| Surgery |  |  |  |
| No | 1 |  |  |
| Yes | 0.38 | 0.36-0.41 | <0.001 |
| Comorbidities |  |  |  |
| Diabetes |  |  |  |
| No | 1 |  |  |
| Yes | 1.13 | 1.07-1.19 | <0.001 |
| Hyperlipidemia |  |  |  |
| No | 1 |  |  |
| Yes | 0.84 | 0.80-0.88 | <0.001 |
| Chronic renal disease |  |  |  |
| No | 1 |  |  |
| Yes | 1.17 | 1.05-1.32 | 0.006 |
| Smoking related lung cancer |  |  |  |
| No | 1 |  |  |
| Yes | 1.01 | 0.91-1.13 | 0.831 |
| Medication use prior to adenocarcinoma diagnosis |  |  |  |
| Inhaled corticosteroids | 1.13 | 1.04-1.24 | 0.007 |
| Oral corticosteroids | 1.21 | 1.16-1.27 | <0.001 |
| Aspirin | 1.13 | 1.08-1.18 | <0.001 |
| Statin | 0.98 | 0.92-1.05 | 0.563 |
| Geographical area |  |  |  |
| Taipei | 1 |  |  |
| North | 0.97 | 0.91-1.03 | 0.336 |
| Central | 0.96 | 0.90-1.01 | 0.139 |
| South | 1.07 | 1.01-1.14 | 0.021 |
| Kaohsiung-Pingtung | 0.99 | 0.94-1.05 | 0.838 |
| East | 1.14 | 1.01-1.28 | 0.035 |
| Urbanization |  |  |  |
| Urban | 1 |  |  |
| Sub-urban | 1.09 | 1.04-1.13 | <0.001 |
| Rural | 1.10 | 1.03-1.16 | 0.004 |
| CI, confidence interval; COPD, chronic obstructive pulmonary disease; HR, hazard ratio; TB, tuberculosis. | | | |

| **Table S3**. Estimated Hazard Ratios for All-Cause Mortality Related to Pulmonary Diseases in Men with Lung Adenocarcinoma Stratified by Stage | | | | | | | | | | | | | | | | |  |
| --- | --- | --- | --- | --- | --- | --- | --- | --- | --- | --- | --- | --- | --- | --- | --- | --- | --- |
|  | All | |  | Stage I+II | | | |  | Stage III | | | |  | Stage IV | | | |
|  | HR (95% C.I.) | p-value |  | HR (95% C.I.) | | p-value | |  | HR (95% C.I.) | | p-value | |  | HR (95% C.I.) | | p-value | |
| **Model 1** |  |  |  | |  | |  | | |  | |  | | |  | |  |
| Asthma (Ref.: Non-asthmatic) | **1.13** (1.05-1.22) | 0.002 | 1.06 (0.80-1.40) | | 0.685 | | 1.13 (0.98-1.32) | | | 0.104 | | **1.14** (1.04-1.26) | | | 0.008 | |  |
| COPD (Ref.: No COPD) | 0.98 (0.92-1.04) | 0.409 | 1.12 (0.91-1.38) | | 0.303 | | 0.96 (0.85-1.07) | | | 0.449 | | 0.97 (0.90-1.05) | | | 0.453 | |  |
| TB (Ref.: No TB) | **1.34** (1.18-1.53) | <0.0001 | **1.69** (1.10-2.58) | | 0.016 | | **1.48** (1.14-1.93) | | | 0.004 | | **1.27** (1.08-1.49) | | | 0.003 | |  |
| **Model 2** |  |  |  | |  | |  | | |  | |  | | |  | |  |
| None (Ref.) | 1 |  | 1 | |  | | 1 | | |  | | 1 | | |  | |  |
| Only asthma | 1.05 (0.93-1.19) | 0.394 | 1.02 (0.66-1.58) | | 0.932 | | 0.87 (0.68-1.11) | | | 0.253 | | 1.13 (0.98-1.31) | | | 0.103 | |  |
| Only COPD | 0.95 (0.89-1.02) | 0.131 | 1.13 (0.89-1.43) | | 0.318 | | 0.88 (0.77-1.01) | | | 0.068 | | 0.96 (0.88-1.04) | | | 0.337 | |  |
| Only TB | **1.28** (1.01-1.63) | 0.043 | **3.11** (1.58-6.11) | | 0.001 | | 1.33 (0.81-2.18) | | | 0.261 | | 1.16 (0.86-1.57) | | | 0.338 | |  |
| Asthma+ COPD | **1.15** (1.04-1.27) | 0.007 | 1.27 (0.91-1.79) | | 0.161 | | **1.22** (1.01-1.47) | | | 0.035 | | 1.12 (0.99-1.27) | | | 0.082 | |  |
| Asthma+TB | 1.47 (0.88-2.44) | 0.140 | 1.08 (0.15-7.9) | | 0.943 | | 1.92 (0.85-4.35) | | | 0.116 | | 1.36 (0.68-2.73) | | | 0.385 | |  |
| COPD+TB | **1.35** (1.12-1.63) | 0.002 | 1.57 (0.8-3.07) | | 0.190 | | 1.34 (0.91-1.98) | | | 0.133 | | **1.32** (1.05-1.66) | | | 0.019 | |  |
| Asthma +COPD+TB | **1.45** (1.12-1.89) | 0.005 | 1.52 (0.59-3.91) | | 0.387 | | 1.86 (0.98-3.52) | | | 0.057 | | **1.39** (1.03-1.89) | | | 0.033 | |  |
| **Model 3** |  |  |  | |  | |  | | |  | |  | | |  | |  |
| None (Ref.) | 1 |  | 1 | |  | | 1 | | |  | | 1 | | |  | |  |
| Any one lung disease | 0.98 (0.92-1.04) | 0.559 | 1.16 (0.94-1.44) | | 0.171 | | 0.90 (0.79-1.01) | | | 0.069 | | 1.00 (0.93-1.08) | | | 0.992 | |  |
| Any two lung diseases | **1.19** (1.09-1.30) | <0.001 | 1.33 (0.97-1.81) | | 0.074 | | **1.26** (1.06-1.49) | | | 0.007 | | **1.16** (1.04-1.30) | | | 0.010 | |  |
| Asthma +COPD+TB | **1.46** (1.12-1.89) | 0.005 | 1.56 (0.61-4.01) | | 0.355 | | 1.87 (0.99-3.53) | | | 0.055 | | **1.40** (1.03-1.89) | | | 0.032 | |  |
| Each model was adjusted for age, low income, surgery, comorbidities, geographical area, urbanization, and medications.  Abbreviation: CI, confidence interval; COPD, chronic obstructive pulmonary disease; HR, hazard ratio; TB, pulmonary tuberculosis. | | | | | | | | | | | | | | | | |  |

| **Table S4.** Estimated Hazard Ratios for All-Cause Mortality Related to Pulmonary Diseases in Women with Lung Adenocarcinoma Stratified by Stage | | | | | | | | | | | | | | | | |
| --- | --- | --- | --- | --- | --- | --- | --- | --- | --- | --- | --- | --- | --- | --- | --- | --- |
|  | All | | |  | | Stage I+II | |  | | Stage III | |  | | Stage IV | |  |
|  | HR (95% C.I.) | p-value | |  |  | HR (95% C.I.) | p-value |  |  | HR (95% C.I.) | p-value |  |  | HR (95% C.I.) | p-value |  |
| **Model 1** |  | |  | |  | |  | |  | |  | |  | |  | |
| Asthma (Ref.: non-asthmatic) | 1.03 (0.93-1.13) | | 0.596 | | **1.41** (1.00-1.99) | | 0.048 | | 1.05 (0.85-1.28) | | 0.661 | | 0.98 (0.87-1.10) | | 0.723 | |
| COPD (Ref.: No COPD ) | 0.96 (0.89-1.05) | | 0.380 | | 0.92 (0.68-1.24) | | 0.563 | | 0.90 (0.76-1.06) | | 0.205 | | 0.99 (0.90-1.10) | | 0.854 | |
| TB (Ref.: No TB) | 1.03 (0.83-1.26) | | 0.813 | | 1.07 (0.52-2.20) | | 0.865 | | 0.98 (0.64-1.51) | | 0.935 | | 1.06 (0.82-1.37) | | 0.648 | |
| **Model 2** |  | |  | |  | |  | |  | |  | |  | |  | |
| None (Ref.) | 1 | |  | | 1 | |  | | 1 | |  | | 1 | |  | |
| Only asthma | 0.94 (0.82-1.07) | | 0.347 | | 1.15 (0.71-1.87) | | 0.578 | | 0.89 (0.67-1.18) | | 0.406 | | 0.94 (0.80-1.09) | | 0.403 | |
| Only COPD | 0.92 (0.84-1.02) | | 0.102 | | 0.82 (0.57-1.18) | | 0.291 | | 0.85 (0.71-1.03) | | 0.104 | | 0.96 (0.86-1.08) | | 0.492 | |
| Only TB | 0.97 (0.67-1.4) | | 0.875 | | 0.65 (0.09-4.75) | | 0.673 | | 1.04 (0.46-2.35) | | 0.919 | | 1.01 (0.66-1.54) | | 0.982 | |
| Asthma+ COPD | 1.07 (0.93-1.22) | | 0.349 | | 1.31 (0.82-2.09) | | 0.254 | | 1.06 (0.81-1.39) | | 0.671 | | 1.03 (0.88-1.22) | | 0.713 | |
| Asthma + TB | 1.50 (0.67-3.35) | | 0.323 | | - | | - | | 2.57 (0.63-10.48) | | 0.187 | | 1.58 (0.59-4.22) | | 0.366 | |
| COPD+TB | 0.95 (0.68-1.33) | | 0.762 | | 0.47 (0.12-1.90) | | 0.288 | | 0.74 (0.41-1.31) | | 0.298 | | 1.26 (0.81-1.97) | | 0.303 | |
| Asthma+ TB +COPD | 1.04 (0.71-1.53) | | 0.848 | | **6.94** (2.72-17.71) | | <0.0001 | | 1.15 (0.36-3.62) | | 0.815 | | 0.85 (0.53-1.36) | | 0.498 | |
| **Model 3** |  | |  | |  | |  | |  | |  | |  | |  | |
| None (Ref.) | 1 | |  | | 1 | |  | | 1 | |  | | 1 | |  | |
| Any one lung disease | 0.93 (0.86-1.01) | | 0.074 | | 0.90 (0.66-1.22) | | 0.481 | | 0.87 (0.74-1.02) | | 0.087 | | 0.95 (0.87-1.05) | | 0.334 | |
| Any two lung diseases | 1.06 (0.93-1.20) | | 0.388 | | 1.07 (0.69-1.66) | | 0.767 | | 1.00 (0.79-1.28) | | 0.994 | | 1.06 (0.91-1.24) | | 0.450 | |
| Asthma+ TB +COPD | 1.04 (0.70-1.53) | | 0.850 | | **6.79** (2.66-17.33) | | <0.0001 | | 1.13 (0.36-3.56) | | 0.836 | | 0.85 (0.54-1.36) | | 0.499 | |
| Each model was adjusted for age, low income, surgery, comorbidities, geographical area, urbanization, and medications.  Abbreviation: CI, confidence interval; COPD, chronic obstructive pulmonary disease; HR, hazard ratio; TB, pulmonary tuberculosis. | | | | | | | | | | | | | | | | |

Supplemental Digital Content

Supplementary Figure S1 illustrates the number of patients with pulmonary diseases at each stage. Model 1, a model containing three pulmonary diseases; Model 3, a count of pulmonary diseases. Abbreviation: COPD = chronic obstructive pulmonary disease, TB = tuberculosis.
